# Misinterpretation of histopathological results as an important risk factor for unneeded surgery – case report of a "near miss" event in a pregnant woman

**DOI:** 10.1186/1754-9493-2-14

**Published:** 2008-06-05

**Authors:** Sigbjørn Løes, Knut Tornes

**Affiliations:** 1 Department of Oral and Maxillofacial Surgery, Haukeland University Hospital, Jonas Lies vei 65, N-5021 Bergen, Norway; 2 Dept. of Oral Surgery and Oral Medicine, Faculty of Medicine and Dentistry, University of Bergen, Årstadvn. 17, N-5009 Bergen, Norway

## Abstract

The oral cavity may exhibit a vast number of pathologic conditions, often dealt with by different medical disciplines. Combined with a substantial variation in clinical appearance, an accurate diagnosis may provide difficult to establish in selected cases. Histopathological investigations are therefore mandatory for correct diagnosis and adequate treatment. We describe a common, truly benign condition in the oral cavity, which due to histopathological misinterpretation was planned for major surgery and subsequent chemotherapy. This was avoided by spontaneous regression of the lesion. The case illustrates that uncritical trust in laboratory diagnostic tests may lead to severe mistreatment.

## Background

The oral cavity may exhibit a myriad of expansive lesions, including cysts, reactive lesions, and benign and malignant neoplasms. Additional laboratory examinations are therefore often used to specify the diagnosis. However, the clinical impact of errors committed in the laboratory is poorly described [1,2]. Recent data suggest that errors in cancer diagnosis may occur in more than 10% of reviewed specimens [1]. We report a case illustrating that modern diagnostic tools in some cases may confuse and mislead, rather than clarify a certain condition.

## Case report

**Figure 1:**
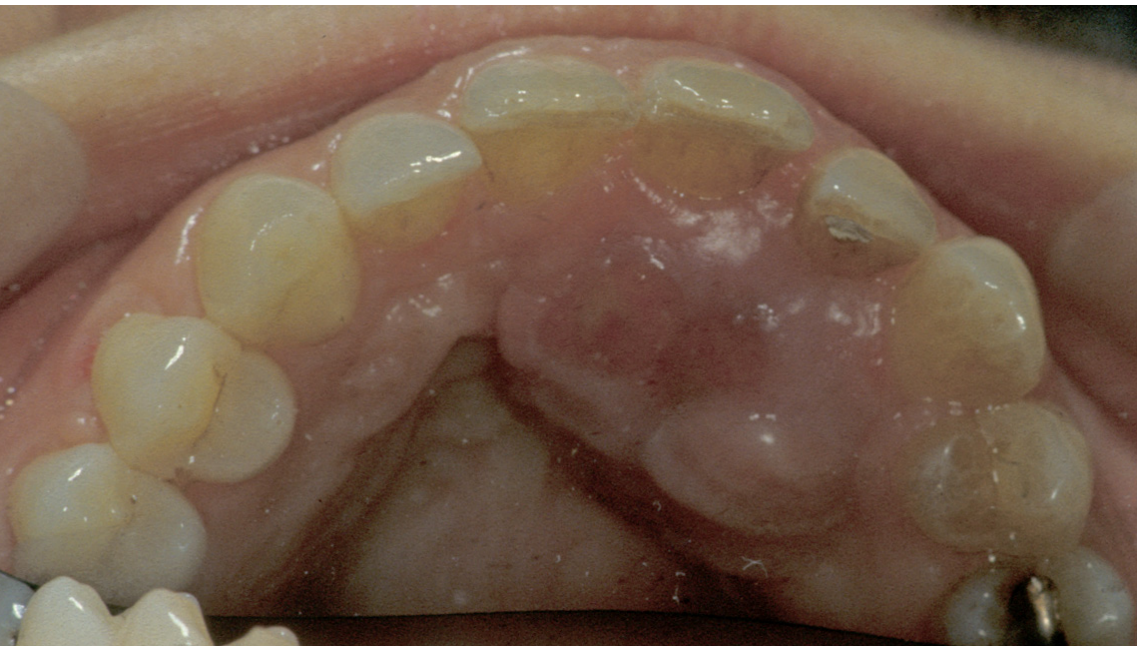
The clinical presentation of the tumour (in mirror).

**Figure 2:**
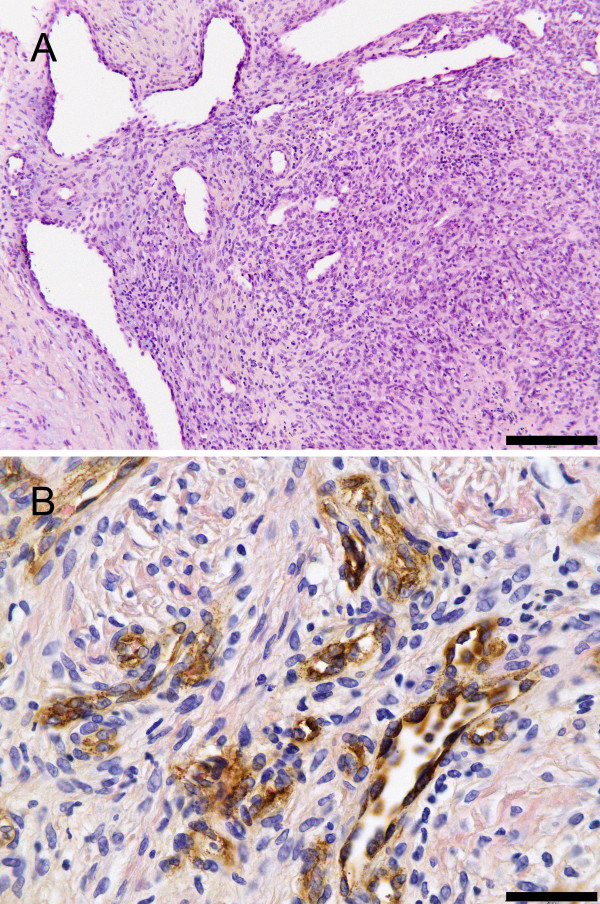
**A: Histopathological appearance of the tumour Hematoxylin-Eosin 10× magnification. **Scalebar: 200 μm. B: Immunohistochemical staining for Factor VIII indicating blood vessel proliferation. Hematoxylin-Eosin. DAB-stained avidin-biotin peroxidase reaction. 40× magnification. Scalebar: 50 μm.

**Figure 3:**
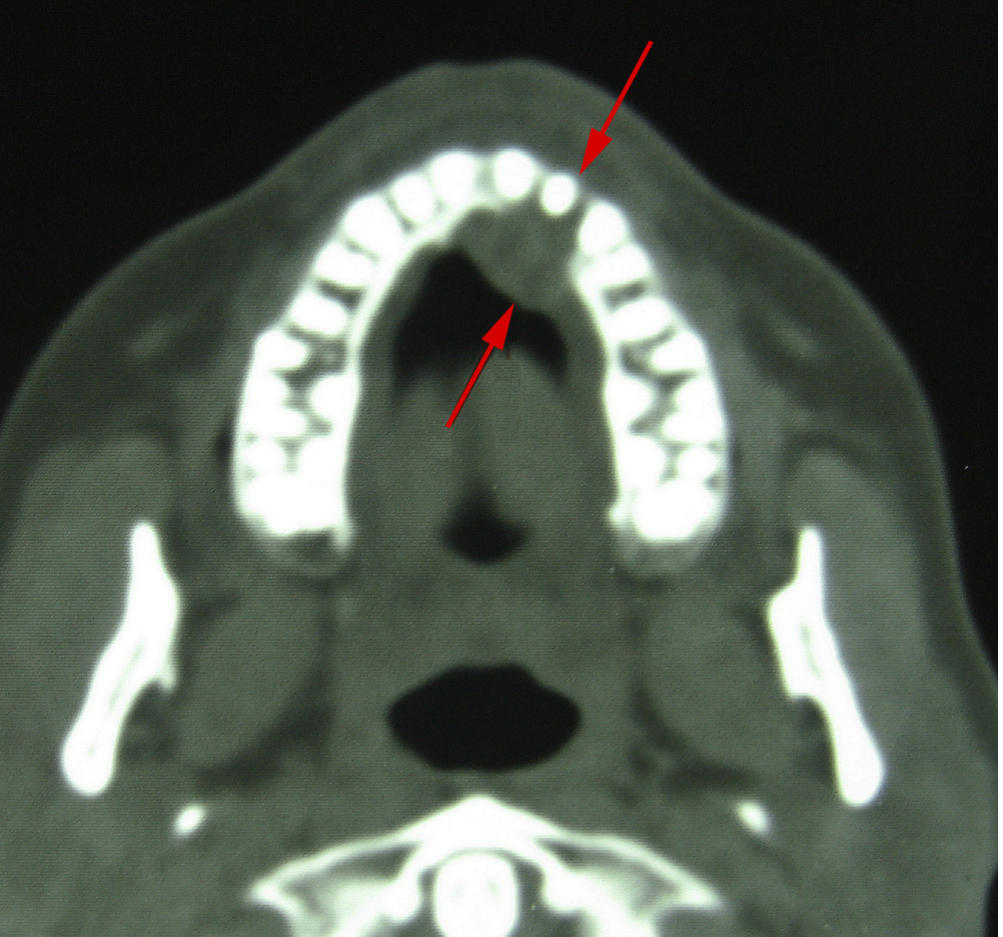
Axial CT scan of the maxilla showing the tumour and destruction of the alveolar process palatinal to tooth 22 (Arrow).

A woman aged 31 years experienced in her fifth, and only successful, in vitro fertilization attempt, after approx. 20 weeks of pregnancy a growing tumour palatinally to tooth 22 (Figure 1). She also noticed that her upper front teeth loosened, and considerable spontaneous bleeding occurred from the tumour. Dental X-rays showed an associated osteolytic process. She was referred to the hospital maxillofacial surgery dept. where the lesion, although somewhat atypical, was clinically recognised as a pyogenic granuloma, a condition not uncommon in the gingiva during pregnancy. The loose teeth could be explained by a possible necrotic tooth with an apical infection, and this tooth was therefore subject to root canal treatment without any effect. The lesion was excised, and remarkably, the histopathologic investigation diagnosed, with considerable uncertainty, an angiosarcoma, or possibly a Kaposis's sarcoma. Staining for Reticulin and immunohistochemical investigation, although of variable quality, showed positive Factor VIII (Figure 2) and Mac 387 (anti-human-myeloid/histiocyte antigen), indicating endothelium and blood vessel proliferation [3]. Hemosiderin staining and immunohistochemical analyses for Lymphocyte common antigen, Keratin and S-100, the latter showing crista neuralis-derived cells, were negative. Due to the possibility of a Kaposi's sarcoma, the patient was HIV tested with a negative result. CT scan showed destruction of the alveolar crest in the actual area, and a soft-tissue tumour approx. 2 cm in diameter (Figure 3). Skeletal scintigraphy showed increased Tc uptake in the actual area. A comprehensive treatment plan was lined up, including resection of the maxilla. As part of the preparations for tumour treatment, the patient delivered short time later two healthy twin boys by caesarean section.

A few days after delivery, just before surgery was to be executed, the palatinal tumour spontaneously regrediated, and disappeared totally within weeks. Her teeth also fastened. The histopathologic diagnosis, unrelated to the clinical development, had at this point been revised to a possible hemangiopericytoma or a hemangiopericytoma-like lesion.

Two years later, the patient again became pregnant (extrauterine), and after about a month, a new tumour, similar to the previous and at the very same position, occurred. This also disappeared spontaneously after abortion.

## Discussion

Pyogenic granuloma is considered a reactive lesion, quite common in the oral cavity. It may clinically mimic several other lesions, including malignancies. The so-called pregnancy tumour is a pyogenic granuloma that seems to appear more easily in the gingiva during pregnancy due to hormonal influence. It can be fast-growing and reach a considerable size. After delivery or termination of pregnancy, they most often disappear spontaneously. The histopathology shows proliferating endothelial cells, the stroma is fibrillar, often arranged in lobular aggregates [4]. The actual sections showed collagenous connective tissue with several blood vessels and a cell-rich area with diffuse cell borders and several mitotic cells. Despite the well-known clinical development of the lesion among the involved pathologists, later re-examinations have not been conclusive concerning histologic diagnosis, and the histopathological appearance seems not typical for any of the previously described entities. Nor is the CT scan typical for a pyogenic granuloma. Recent advances have identified antigens that may help distinguish vasoproliferative tumours [3,5,6], and it is likely that in the future, molecular techniques may play an even more important role in tumour diagnostics and also subsequent choice of treatment [7]. Medical professionals, pathologists included, will probably still commit mistakes, particularly when deprived of or misleaded by *e.g. *insufficient clinical data [8]. To detect and avoid such errors, different audit regimes have been proposed [9]. In this particular case, most of the recommended audit strategies [9] were performed. Sarcomas and other relatively rare malignancies are at the Dept. of pathology dealt with by a dedicated group of pathologists, and foreign experts are consulted in special cases, including this one. There are also regular organised discussion meetings between pathologists and surgeons for all malignant tumours to avoid mistakes based on misinterpretation of clinical versus histological data.

The clinical course in this case is highly consistent with a pregnancy tumour. It is, to our best knowledge, not been reported that neither angiosarcomas nor hemangiopericytomas may undergo spontaneous regression. However, the possibility for hormonal and endothelial growth factor influence in vascular tumours during pregnancy is discussed [10,11].

The patient has to this day not experienced any later recurrences of the lesion. Her two twin boys delivered as preparation for tumour treatment have also grown up perfectly healthy.

## Conclusion

As medicine in general becomes increasingly dependent of laboratory investigations, sample analyses, and advanced imaging techniques, it is of great importance to emphasise the value of the clinical consultation as well. The case also reminds us that simple conditions may be misinterpreted, possibly causing serious mistreatment.

## Consent

Written informed consent was obtained from the patient for publication of this case report. A copy of the written consent is available for review by the Editor-in-Chief of this journal.

## Declaration of competing interests

The authors declare that they have no competing interests.

## Authors' contributions

KT was responsible for diagnosis and treatment of the patient and has contributed to the manuscript preparation. SL has re-examined the case, contributed to patient follow-up consultations and drafted the manuscript.
